# Pathophysiology of homocysteine: insights into ion channel dysfunction

**DOI:** 10.1007/s00424-026-03165-0

**Published:** 2026-03-31

**Authors:** Nikola Chmúrčiaková, Robin N. Stringer, Leoš Cmarko, Alzbeta Filipova, Lubica Lacinova, Norbert Weiss

**Affiliations:** 1 Institute of Biology and Medical Genetics, First Faculty of Medicine, Charles University, Prague, Czech Republic; 2 Department of Pathophysiology, Third Faculty of Medicine, Charles University, Prague, Czech Republic; 3 Center of Biosciences, Institute of Molecular Physiology and Genetics, Slovak Academy of Sciences, Bratislava, Slovakia

**Keywords:** Homocysteine, Ion channels, Pathophysiology, Cardiovascular disease, Neurological disease

## Abstract

Homocysteine is a non-proteinogenic amino acid formed during the metabolism of methionine to cysteine and plays a critical role in maintaining cellular homeostasis. Although multiple enzymatic pathways tightly regulate homocysteine levels, their dysfunction can lead to elevated circulating homocysteine, which is recognized as a risk factor for various cardiovascular and neurological disorders. While most evidence linking homocysteine to specific pathologies comes from observational studies, emerging data suggest that dysregulation of ion channels may be an important underlying mechanism. In this review, we summarize the effects of homocysteine on the expression and function of key ion channel families including calcium, sodium, and potassium channels, and discuss their potential pathophysiological implications.

## Introduction

**Fig. 1:**
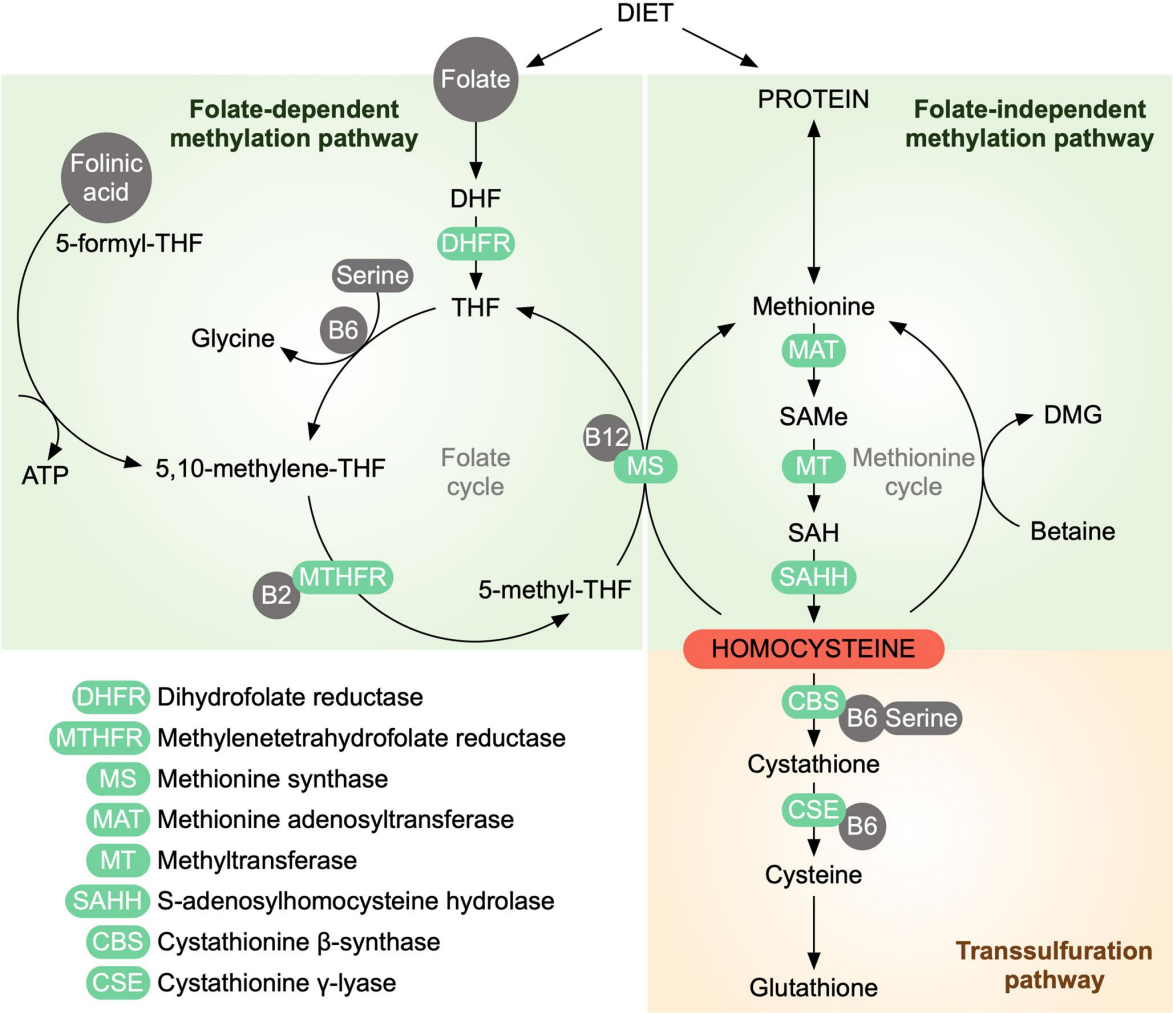
Overview of homocysteine metabolism. Homocysteine occupies a central position in the methionine cycle and is regulated by remethylation and transsulfuration pathways. Folate-dependent remethylation involves the conversion of homocysteine to methionine via methionine synthase (MS) using 5-methyl-THF generated by MTHFR, with vitamin B12 as a cofactor. Methionine is converted to S-adenosylmethionine (SAM), the universal methyl donor, and subsequently to S-adenosylhomocysteine (SAH), which is hydrolyzed back to homocysteine. Homocysteine can alternatively be remethylated via a folate-independent, betaine-dependent pathway or irreversibly metabolized through the transsulfuration pathway to cysteine and glutathione via cystathionine β-synthase (CBS) and cystathionine γ-lyase (CSE)

Homocysteine (Hcy) is a non-proteinogenic, sulphur-containing amino acid that serves as an essential intermediate in the metabolism of methionine to cysteine. In plasma, Hcy predominantly exists in oxidized forms, either bound in disulfides (70–80%) or as a mixed disulfide with other thiols (20–30%), whereas less than 1% is present as free thiol [57]. Although methionine is the sole dietary precursor of Hcy, its concentration is tightly regulated by a series of enzymatic reactions that require cofactors from the vitamin B complex, particularly vitamins B6 and B12, as well as folic acid. Accordingly, Hcy can be recycled back to methionine via the remethylation pathway or irreversibly metabolized to cysteine through the transsulfuration pathway [45] (Fig. 1).

These pathways maintain physiological plasma Hcy concentrations within a reference range of 5–13 µmol/L [108]. Notably, Hcy levels increase with age and are higher in men and postmenopausal women [33, 143]. Disruption of methionine metabolism, resulting from mutations in genes encoding enzymes involved in Hcy metabolism [121] or from deficiencies in essential cofactors such as vitamin B12 [27, 59], leads to elevated plasma Hcy levels, a condition termed hyperhomocysteinemia (HHcy). Depending on severity, HHcy is classified as mild to moderate (15–30 µmol/L) or severe (> 100 µmol/L) [89], and is recognized as an independent risk factor for a range of cardiovascular and neurological disorders [7, 65, 73, 90, 116, 138].

**Fig. 2:**
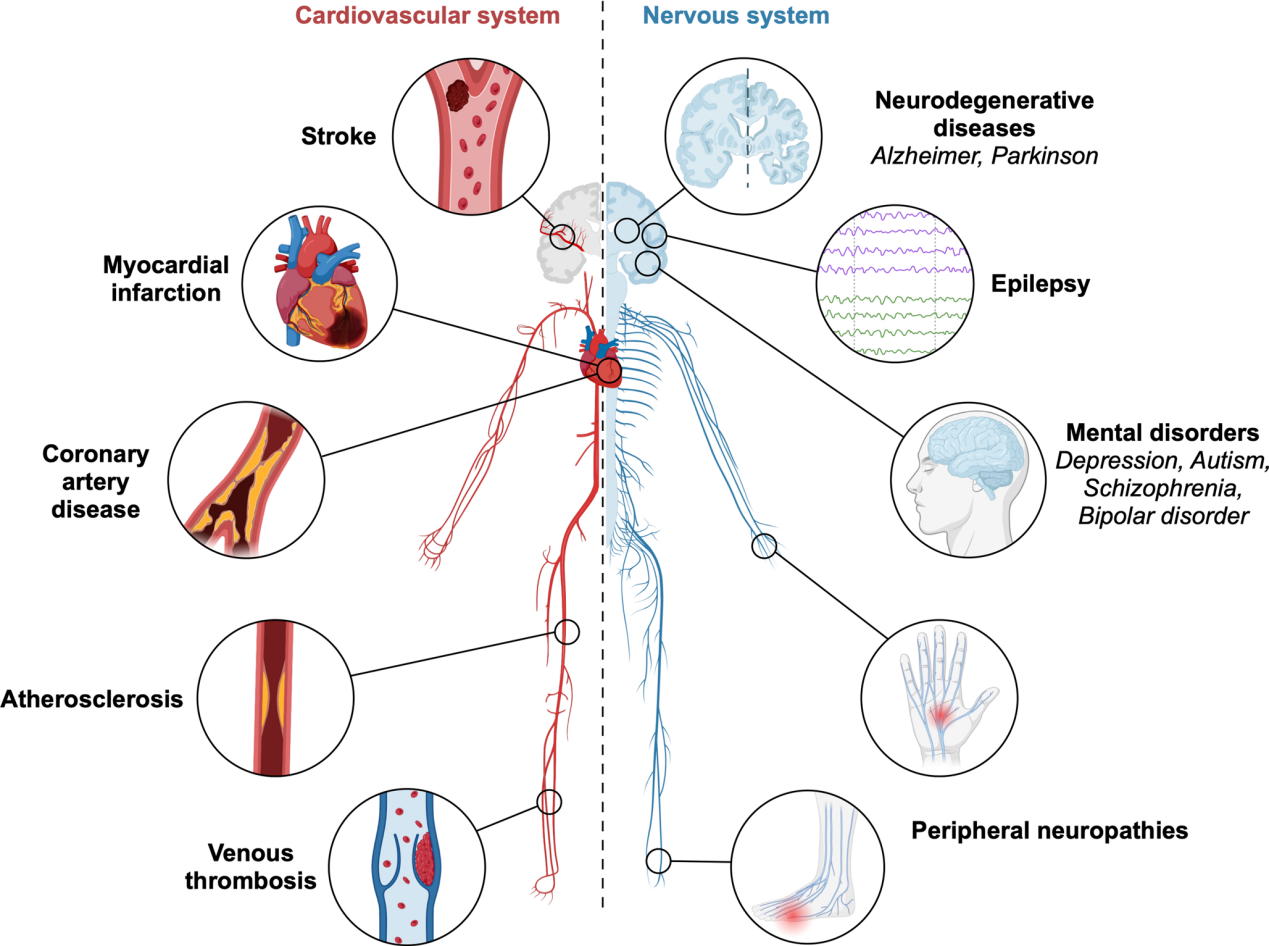
Systemic effects of hyperhomocysteinemia on the cardiovascular and nervous systems. Schematic overview illustrating the major organ systems affected by elevated homocysteine levels. The left side (red) highlights the cardiovascular system, including the heart, large and small blood vessels, and the vascular endothelium, emphasizing homocysteine-associated alterations such as endothelial dysfunction, vascular remodeling, and thrombogenic changes. The right side (blue) depicts the nervous system, including the brain, peripheral nerves, and neuromuscular structures, illustrating the involvement of homocysteine in neurotoxicity, altered neuronal excitability, and peripheral neuropathies. Insets represent selected cellular and tissue-level targets within each system, underscoring the multisystemic impact of hyperhomocysteinemia

Since the first reports of elevated homocysteine levels in humans in 1962 [51, 126], HHcy has attracted sustained interest across multiple scientific disciplines. However, relatively few large-scale epidemiological studies have been conducted, making accurate estimates of its current global prevalence challenging. In 1999, one of the first large population-based studies reported that approximately 5–7% of the general population exhibited mild HHcy [57]. In contrast, more recent studies in the Chinese population suggest substantially higher prevalence rates, ranging from 37.2% [147] to 50.8% [103, 144]. Prevalence rates in other populations vary considerably and are influenced by multiple factors, including advancing age, increased body mass index, smoking status [144], genetic enzyme dysfunction, impaired availability of metabolic cofactors, excessive methionine intake, comorbid diseases, and the use of certain medications [76]. This apparent rise in HHcy prevalence underscores the need for in-depth investigation of the molecular mechanisms underlying its pathophysiological effects, particularly given the growing number of associations reported between HHcy and human disease. HHcy has been linked to a wide range of disorders affecting both the cardiovascular system, such as congestive heart failure and atherosclerosis, and the nervous system, including Parkinson’s disease, Alzheimer’s disease, multiple sclerosis, and epilepsy [62] (Fig. 2).

Elevated Hcy levels have also been associated with osteoporosis, chronic renal failure, hypothyroidism, insulin-resistant diabetes, polycystic ovarian syndrome, and gastrointestinal disorders [3]. These associations raise an important question: what are the molecular mechanisms through which HHcy contributes to the development of such diverse pathologies? From an etiological perspective, dysregulation of ion-permeable channels, including calcium, sodium, and potassium channels, represents a plausible mechanistic link. These channels play essential roles in maintaining cardiovascular and nervous system homeostasis and are frequently targeted by pharmacological therapies [19, 87]. Moreover, these ion channels are key regulators of cellular electrical excitability, and their dysfunction correlates with many neurological and cardiovascular manifestations associated with HHcy. For instance, neurological symptoms include seizures, particularly in inherited forms of HHcy due to cystathionine β-synthase deficiency [10], as well as anxiety [37], depression [11], psychosis, cognitive impairment, and peripheral neuropathy [86]. In parallel, altered excitability of cardiomyocytes contributes to HHcy-associated arrhythmias and dysregulation of vascular tone [92].

In this review, we focus on three major ion channel families: calcium, sodium, and potassium channels. For each family, we first provide a general overview of the channels, then describe how HHcy affects their function across endothelial, cardiac, and neuronal cells, drawing on evidence from both in vivo animal models and in vitro cellular studies.

## Animal models of hyperhomocysteinemia

**Fig. 3:**
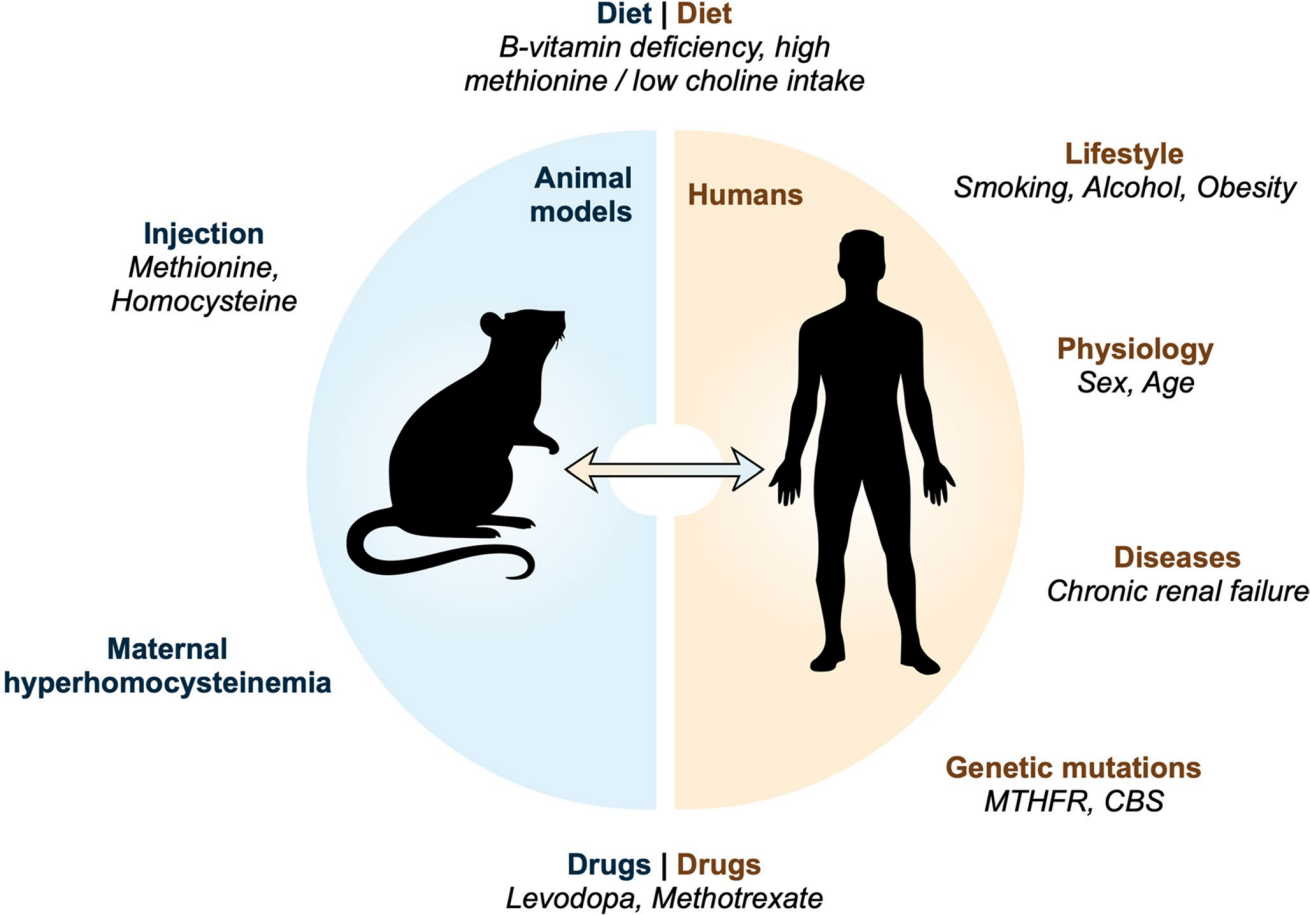
Determinants and experimental models of hyperhomocysteinemia. Schematic representation of the major factors contributing to hyperhomocysteinemia (HHcy) in humans (right, green) and the principal strategies used to induce HHcy in animal models (left, orange). In humans, elevated homocysteine levels arise from a combination of dietary factors (e.g. B-vitamin deficiency, increased methionine intake, limited choline), lifestyle influences (smoking, obesity, alcohol consumption), physiological variables (age, sex), pathological conditions (such as chronic renal failure), genetic mutations affecting one-carbon metabolism (e.g. *MTHFR*, *CBS*), and pharmacological treatments (e.g. levodopa, methotrexate). In experimental animals, HHcy is commonly induced through dietary manipulation, parenteral administration of methionine or homocysteine, maternal HHcy, or drug treatment. The figure highlights the conceptual parallels between clinical and experimental determinants of HHcy

Animal models are essential tools for investigating the molecular mechanisms underlying human diseases associated with HHcy. Numerous approaches have been developed to induce HHcy in animals, each with specific advantages and limitations in recapitulating impaired homocysteine metabolism observed in humans [32] (Fig. 3).

The most common method involves dietary manipulation of one-carbon (C1) metabolism, typically through diets deficient in B vitamins, especially B6, B12, and folic acid, or by L-methionine overconsumption [12, 25, 64]. In some studies, homocysteine itself or methyl group acceptors that interfere with C1 metabolism are administered directly to animals to elevate systemic Hcy levels [101]. Dietary approaches provide continuous exposure, mimicking chronic HHcy conditions.

An alternative strategy employs parenteral administration of homocysteine, usually via subcutaneous or intraperitoneal injection [78]. Unlike dietary methods, the chronicity and intensity of HHcy depend on injection frequency and dosage, offering a more controlled temporal elevation of Hcy.

Genetic approaches represent another major category of animal model. Mutations are introduced into genes encoding key enzymes in C1 metabolism, most notably cystathionine beta-synthase (CBS), which converts homocysteine to cystathionine in a vitamin B6-dependent manner [2, 131]. Disruption of CBS function leads to Hcy accumulation, recapitulating HHcy observed in humans with CBS deficiency [40]. Similarly, methylenetetrahydrofolate reductase (MTHFR), required for vitamin B12-dependent remethylation of homocysteine to methionine, has been targeted to generate genetic models of HHcy [2, 26].

Some studies focus on the maternal HHcy effect, where elevated Hcy levels in pregnant females result in increased exposure in their offspring, either through the placenta or via lactation. By increasing maternal homocysteine before, during, or after pregnancy, Hcy crosses the placenta or is transferred via breast milk, affecting developing embryos and neonates without directly manipulating the pups [141]. This approach allows investigation of developmental and early-life effects of HHcy.

A minority of studies combine multiple methods to induce HHcy, such as pairing dietary manipulation with genetic modifications, or employ alternative strategies designed to simulate human lifestyle influences. In addition, primary cultures of animal cells treated with Hcy have been widely used to study cellular and molecular effects in a controlled in vitro environment [44, 107].

Collectively, these animal and cellular models provide a robust framework for exploring the mechanistic consequences of HHcy, particularly its modulation of calcium, sodium, and potassium channels in endothelial, cardiac, and neuronal cells, which are discussed in the following sections.

## Calcium channels

### General overview

Calcium channels constitute a major superfamily of proteins that allow the influx of calcium ions from both intracellular stores and the extracellular milieu [8, 28]. Calcium influx plays a dual role: it contributes to cellular excitability by enhancing positive charge inside the cell, and it acts as a second messenger regulating numerous signaling cascades [23, 146]. Through these mechanisms, calcium channels maintain intracellular calcium homeostasis and tightly control several cellular processes, including heart and muscle contractions, neurotransmission, learning and memory, embryonic formation and development, cell proliferation and apoptosis, cell division and differentiation, energy metabolism, protein phosphorylation and dephosphorylation, and gene expression and regulation [112, 132, 145].

Two families of calcium-permeable channels play a role in HHcy: voltage-gated calcium channels (VGCCs, Ca_v_) [21, 24, 42, 146] and ligand-gated calcium channels (LGCCs). Voltage-gated calcium channels comprise several subtypes that are commonly classified according to their activation properties into high-voltage-activated (HVA) channels, which require strong membrane depolarization, and low-voltage-activated (LVA) channels, which open at comparatively more negative membrane potentials. Among VGCCs, high-voltage-activated L- and P/Q-types, and low-voltage-activated T-type channels are particularly relevant to HHcy, whereas other VGCCs have not been documented. HVA L-type VGCCs are characterized by slow activation and inactivation kinetics and play pivotal roles in several organ systems, including the smooth muscle of the intestine and vasculature, the heart, and the central nervous system (CNS). In neurons, L-type channels contribute to excitability, neurotransmitter release, learning, and memory. Dysregulation of these channels has been linked to various cardiac and neuronal disorders, including Timothy syndrome, hypokalemic periodic paralysis, and bipolar affective disorder [20]. L-type channels carry calcium influx predominantly during the plateau phase of the action potential (AP), a contribution that is particularly pronounced in cardiomyocytes and also affects the frequency of AP firing in neuronal cells [9].

P/Q-type calcium channels are another class of HVA channels, highly expressed in the CNS, predominantly in cerebellar Purkinje and granule cells. They are primarily localized in presynaptic terminals, where they mediate fast neurotransmission through vesicular exocytosis driven by calcium influx [38], but they also influence postsynaptic calcium signaling in Purkinje cells [139]. The *CACNA1A* gene encoding P/Q channels undergoes extensive alternative splicing, producing a wide array of P/Q channels with distinct electrophysiological properties [13]. Dysregulation of P/Q channels has been associated with disorders such as episodic ataxia type 2 (EA2), spinocerebellar ataxia type 6 (SCA6), and familial hemiplegic migraine-1 (FHM1) [70, 96, 136, 137].

T-type channels, classified as LVA channels, exhibit fast activation and inactivation kinetics and a low activation threshold near the resting membrane potential of many neurons [100, 133, 135]. These channels are widely distributed throughout the body, including the brain, heart, muscles, endocrine cells, and bones. They play crucial roles in regulating membrane oscillations and pacemaking [67], initiating low-threshold calcium spikes and AP bursts in neuronal cells, and mediating hormone secretion. Dysregulation of T-type channels has been implicated in several pathophysiological conditions, including pain, atrial fibrillation, epilepsy, hypertension, cancer, and congestive heart failure, although most of these disorders involve multiple contributing factors beyond T-type channel activity [68, 134].

In addition to VGCCs, a diverse group of ligand-gated and receptor operated channels can also mediate calcium entry. These include ionotropic glutamate receptors such as NMDA receptors [39], as well as other calcium-permeable channels including acid-sensing ion channels (ASICs) [104] and several members of the transient receptor potential (TRP) channel family [148], which contribute to neuronal excitability and sensory signaling in multiple physiological and pathological contexts, such as in the pain pathways [14]. Ligand-gated calcium channels, notably NMDA receptors of the glutamate receptor family, mediate excitatory neurotransmission in the CNS by allowing the influx of sodium and calcium ions, triggering depolarization that activates voltage-gated sodium channels and AP firing. NMDA receptors are extensively expressed throughout brain development and are central to normal brain function, including neuronal development and synaptic plasticity [98]. Consequently, alterations in NMDA receptor activity have been associated with neurological and psychiatric disorders such as Alzheimer’s disease, ischemic stroke, epilepsy, schizophrenia, and mood disorders, many of which overlap with conditions linked to HHcy [30].

**Table 1:**
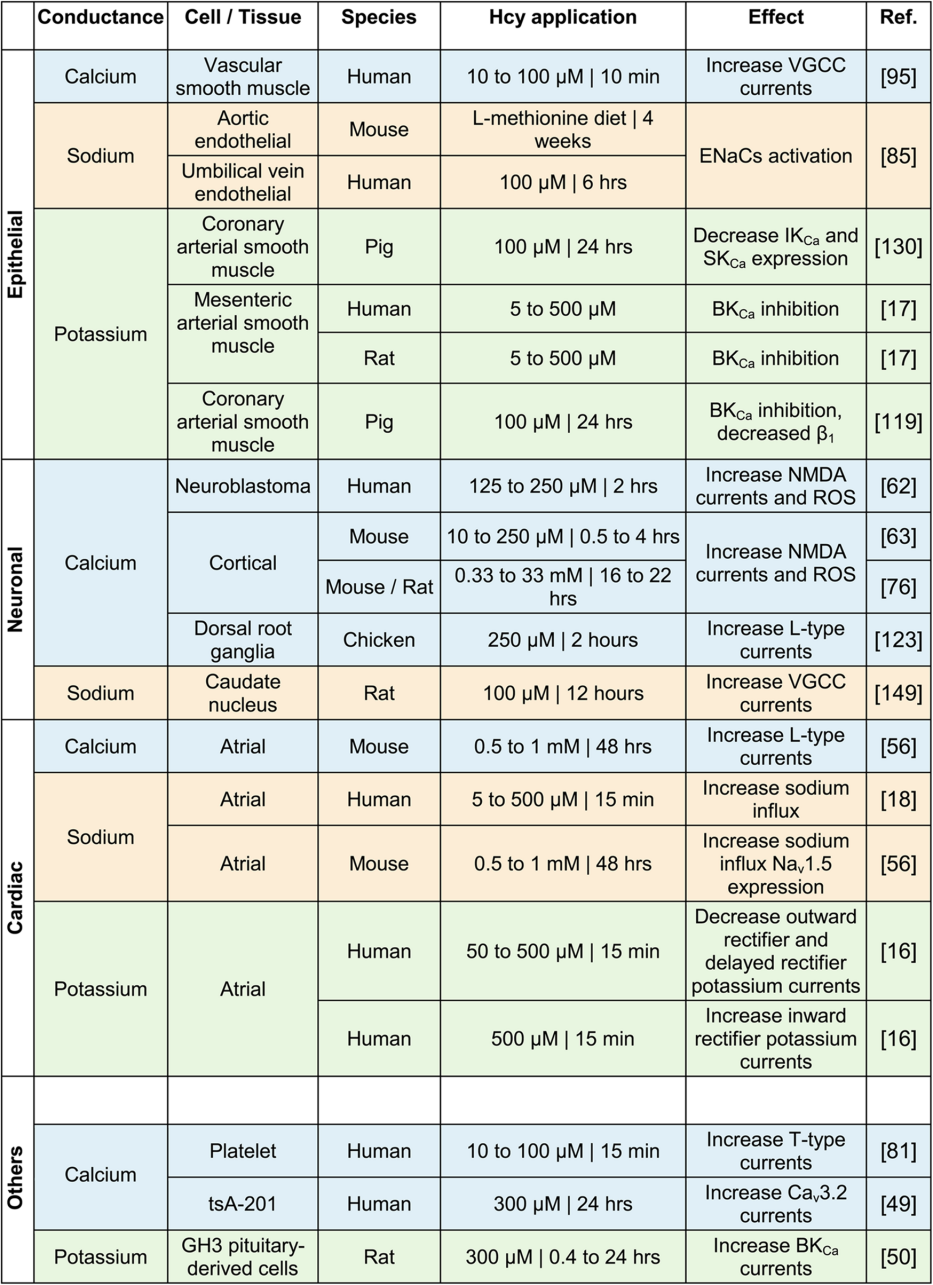
Effects of homocysteine on ion channels across different cell types

Summary of reported effects of homocysteine on calcium (blue), sodium (beige), and potassium (green) channels in epithelial, neuronal, and cardiac cell types

### Effects of HHcy on calcium channels

#### Endothelial cells

**Fig. 4:**
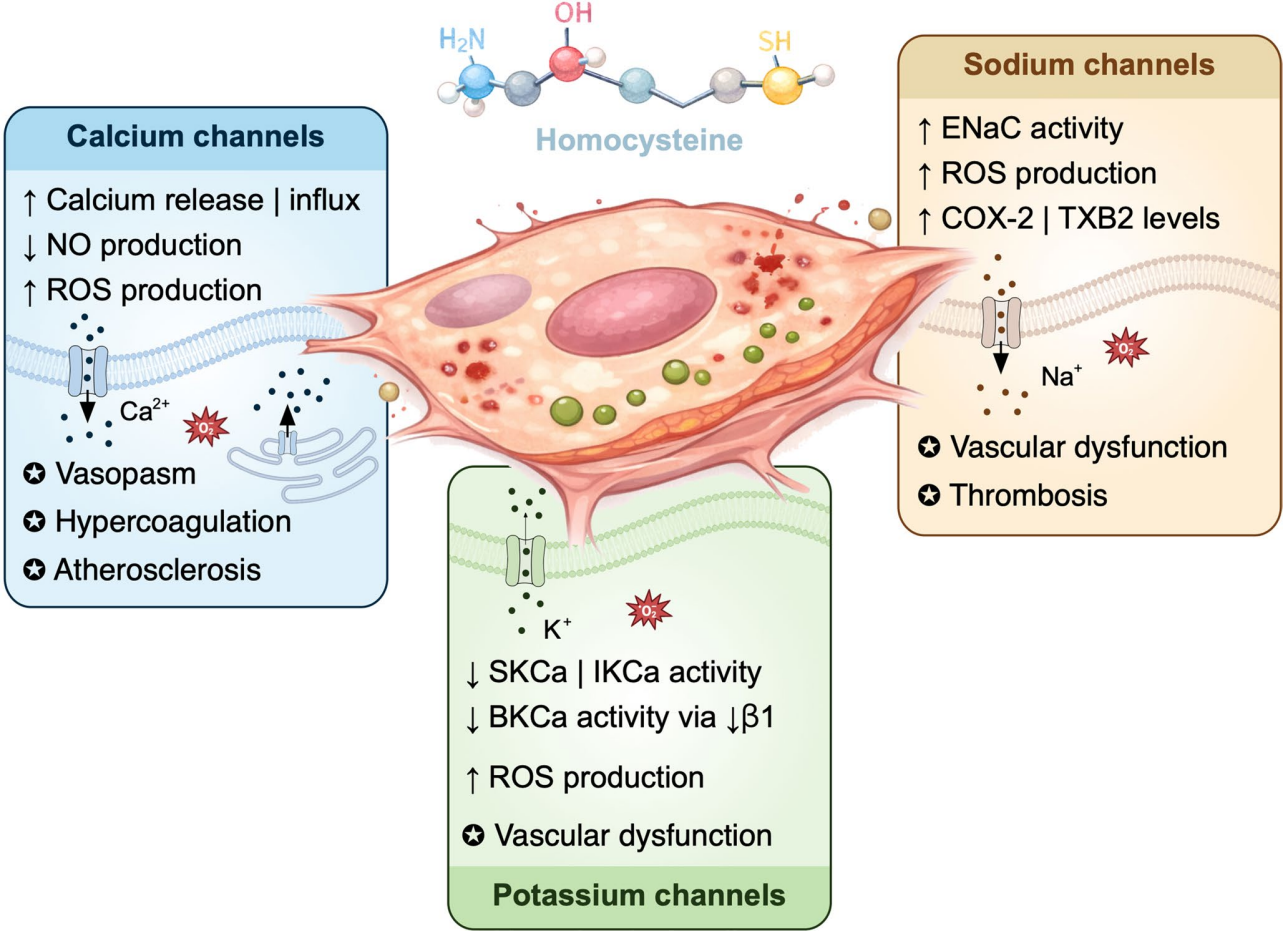
Effects of homocysteine on ion channels in endothelial cells. Schematic overview illustrating the mechanisms by which elevated homocysteine (Hcy) disrupts endothelial ion channel activity and promotes vascular dysfunction. Hcy increases intracellular calcium levels by stimulating calcium release from intracellular stores and enhancing calcium influx through voltage-gated calcium channels, which contributes to reactive oxygen species (ROS) production, suppression of nitric oxide (NO) synthesis, vasospasm, and atherosclerosis. Hcy also activates epithelial sodium channels (ENaCs), leading to impaired endothelium-dependent relaxation through ROS generation and activation of cyclooxygenase-2 (COX-2) and thromboxane signaling pathways. In addition, Hcy inhibits calcium-activated potassium channels (SK_Ca_​, IK_Ca_​, and BK_Ca_) through mechanisms involving oxidative stress and endoplasmic reticulum stress, including downregulation of the BK_Ca_​ β_1_ subunit. The combined dysregulation of these ion channels contributes to endothelial dysfunction, vascular hyperreactivity, thrombosis, and increased risk of cerebrovascular complications such as ischemic stroke and vascular dementia

HHcy profoundly affects calcium homeostasis in endothelial cells and vascular smooth muscle cells (VSMC) (Fig. 4; Table 1).

In VSMCs from healthy rats, Hcy induces a transient rise of intracellular calcium primarily through a release of intracellular calcium stores, such as the endoplasmic reticulum (ER). The suggested mechanism involves the activation of the phosphoinositide signaling pathway by Hcy followed by IP3 (inositol 1,4,5-trisphosphate)-mediated calcium release [93]. Similar effects have been observed in human umbilical VSMCs, where, in contrast, Hcy enhances extracellular calcium influx through voltage-gated calcium channels, contributing to vasospasm and the suppression of endothelial nitric oxide (NO) synthesis [95]. This increase in cytosolic calcium enhances cellular sensitivity to angiotensin II, which triggers intracellular signaling cascades producing reactive oxygen species (ROS). ROS, in combination with Hcy, further suppress NO production [124], a known vasodilator and anti-thrombogenic factor in vascular endothelial cells [72, 102]. These mechanisms play a major role in promoting hypercoagulation and atherosclerosis, with studies demonstrating a positive correlation between plasma Hcy levels and the progression of atherosclerotic lesions [115]. In human endothelial progenitor cells, elevated Hcy induces apoptosis and ER stress-mediated caspase-3 activation, accompanied by calcium release from intracellular stores [83]. Together, these effects explain, at least in part, the contribution of HHcy to vascular dysfunction and suggest potential impacts on neurological disorders involving the vascular system, such as ischemic stroke, headaches, and vascular dementia.

#### Cardiac cells

**Fig. 5:**
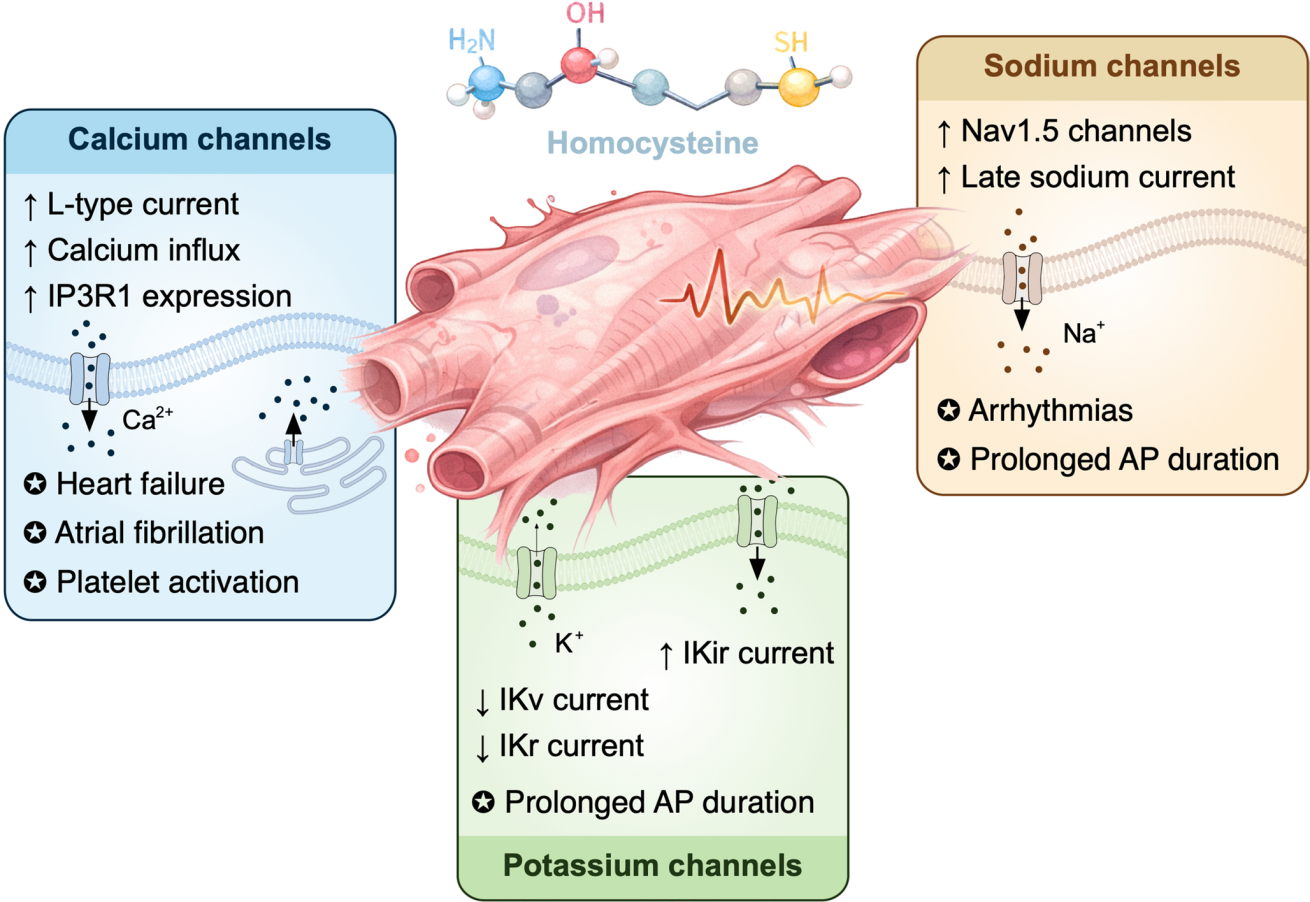
Effects of homocysteine on ion channels in cardiac cells. Elevated homocysteine (Hcy) levels disrupt cardiac electrophysiology by modulating calcium, sodium, and potassium channel activity in cardiomyocytes. Hcy enhances L-type Ca^2+^ currents and increases intracellular Ca^2+^ levels, partly through upregulation of inositol-1,4,5-trisphosphate receptor type 1 (IP_3_R1), contributing to prolonged action potential (AP) duration and increased susceptibility to atrial fibrillation and heart failure. Hcy also promotes Ca^2+^ influx through T-type channels and stimulates platelet activation, supporting pro-thrombotic mechanisms. In sodium channels, Hcy increases voltage-gated Na^+^ currents, particularly the late sodium current (I_NaL_), through enhanced Na_v_1.5 channel expression and altered channel inactivation kinetics. Increased I_NaL_ leads to membrane depolarization, prolonged AP duration, and secondary Ca^2+^ overload via Na^+^/Ca^2+^ exchanger activity, thereby promoting arrhythmogenesis. Hcy further alters cardiac repolarization by inhibiting voltage-gated outward and delayed rectifier potassium currents while enhancing inwardly rectifying potassium currents (I_K1_). These combined effects disrupt normal repolarization and increase electrical instability. Collectively, homocysteine-induced ion channel dysregulation contributes to cardiac complications including arrhythmias, atrial fibrillation, platelet aggregation, and congestive heart failure

In cardiac tissues, HHcy has been shown to alter calcium dynamics in atrial myocytes (Fig. 5; Table 1).

Mice fed a high Hcy diet exhibit increased L-type calcium current, enhanced late sodium current, and elevated intracellular calcium concentrations [56]. This increase may be partly due to Hcy-induced upregulation of the IP3 receptor type 1 (IP3R1). Prolongation of the AP duration has also been observed, which may elevate the risk of atrial fibrillation. While increased calcium influx contributes to this effect, enhanced late sodium current and inhibition of repolarizing transient outward and delayed rectifier potassium currents also play important roles in prolonging the AP [17]. Moreover, Hcy induces calcium influx through T-type channels in platelets from healthy human volunteers, enhancing platelet activation and aggregation, which may contribute to thrombogenic effects seen in HHcy patients [6, 35, 81]. In cardiac neural crest cells isolated from chick embryos, Hcy triggers IP3-mediated release of calcium from the ER, increasing cell attachment while inhibiting migration, resulting in neurocristopathy [60]. Collectively, these calcium-dependent effects in endothelial cells, VSMCs, and cardiac neural crest cells may contribute to the congestive heart failure frequently observed in patients with HHcy, although this is a multifactorial condition and direct causality remains complex to establish. Nevertheless, plasma Hcy levels serve as a useful diagnostic marker for future cardiac complications [29].

**Fig. 6:**
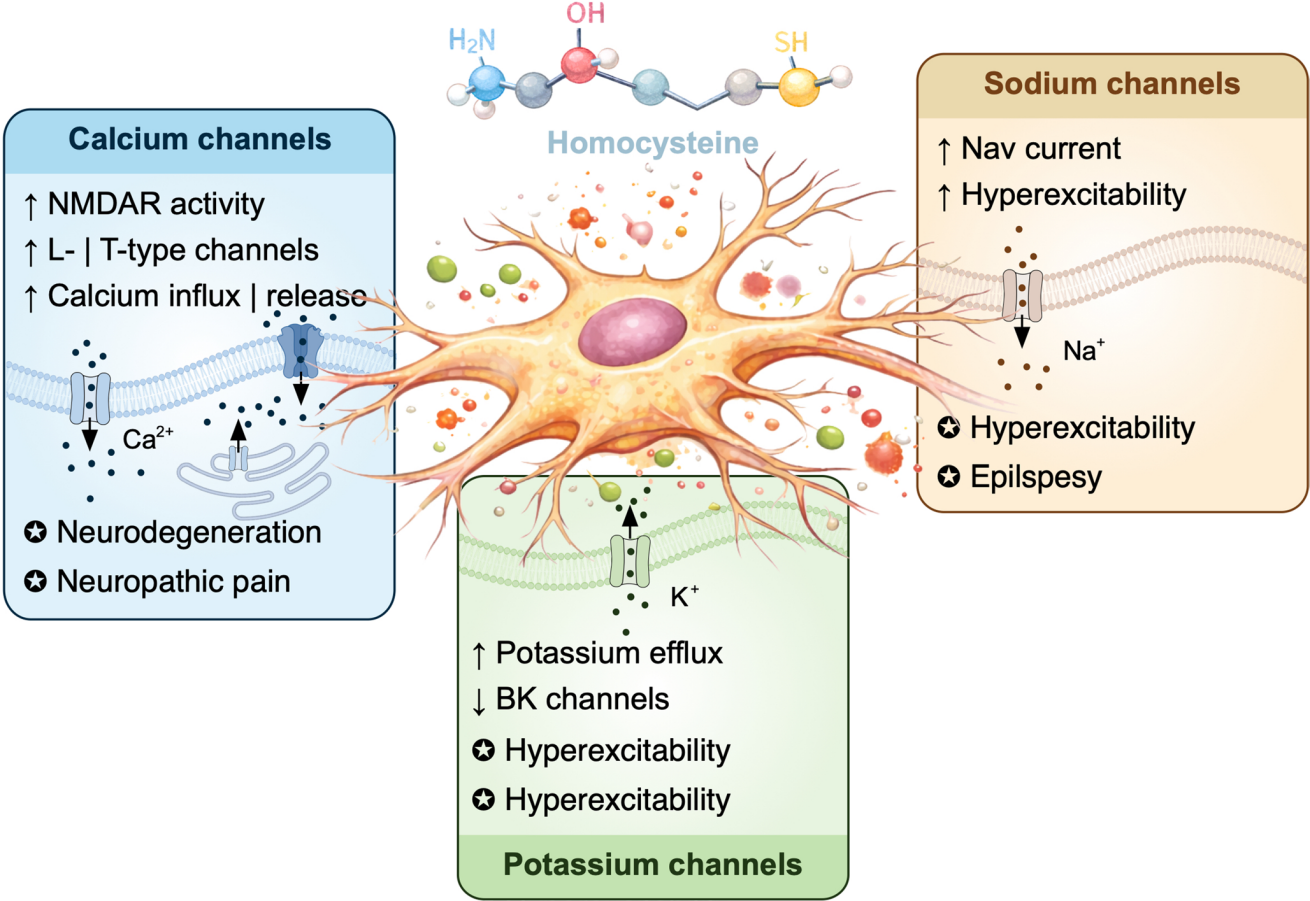
Effect of homocysteine on ion channels in neuronal cells. Schematic overview of the molecular and cellular mechanisms by which elevated homocysteine (Hcy) disrupts neuronal ion channel function. Hcy acts as an agonist of NMDA receptors, promoting calcium influx in cortical and cerebellar neurons, and increases calcium entry through L-type and T-type (Cav3.2) voltage-gated calcium channels, particularly in dorsal root ganglion neurons. Calcium overload triggers reactive oxygen species (ROS) generation, further activating P/Q-type channels and amplifying intracellular Ca2+ dysregulation, leading to oxidative stress and apoptosis. Hcy also enhances voltage-gated sodium channel (VGSC) activity, increasing Na+ currents and shifting channel activation toward more hyperpolarized potentials, thereby promoting neuronal hyperexcitability and excitotoxicity. In addition, Hcy modulates potassium channel activity, including BK channels, potentially altering K+ efflux and impairing action potential repolarization and synaptic signaling. Together, these effects contribute to neuronal dysfunction and are implicated in a wide range of neurological and neurodegenerative disorders associated with hyperhomocysteinemia, including epilepsy, neurodegeneration, neuropathic pain, and cognitive impairment

#### Neuronal cells

In neuronal cells, elevated Hcy concentrations affect both the release of calcium from intracellular stores and extracellular influx (Fig. 6 and Table1)

In cultured human neuroblastoma cells, Hcy directly stimulates calcium influx through NMDA receptors, which leads to neurotoxic effects upon exposure to high concentrations of Hcy [62]. Subsequent studies confirm NMDA receptor involvement in Hcy-mediated calcium signaling, using cortical neurons from embryonic mice and rats [63, 75]. Blockade of NMDA channels with MK-801 prevents this calcium influx, demonstrating that Hcy acts as an agonist. Subunit-specific effects have been observed, with GluN1/2A-composed NMDA receptors preferentially activated in cortical neurons and GluN2C/D-composed receptors in cerebellar neurons [110, 111].

In dorsal root ganglion (DRG) neurons from wild-type chicks, Hcy primarily increases calcium influx through L-type VGCCs possibly by affecting channel trafficking, preceding calcium release from internal stores [123]. This calcium overload promotes ROS generation, which further stimulates P/Q-type voltage-dependent channels, amplifying intracellular calcium overload [94]. Such dysregulation induces oxidative stress and apoptosis in neuronal cells, contributing to neurological disorders associated with HHcy, including age-related macular degeneration, Alzheimer’s disease, Parkinson’s disease, autism, schizophrenia, bipolar disorder, vascular dementia, peripheral neuritis, stroke, multiple sclerosis, epilepsy, and headaches [30, 46, 47, 117].

Through a mechanism involving activation of protein kinase C-dependent signaling pathway, Hcy enhances T-type currents in DRG neurons, by promoting the recycling of Ca_v_3.2 channels back to the plasma membrane [50]. Consequently, in a rat model of prenatal hyperhomocysteinemia, chronic elevation of homocysteine leads to peripheral neuropathy, consistent with the role of Ca_v_3.2 channels in pain transmission. Pharmacological blockade of T-type channels restores normal mechanical sensation, confirming their involvement in Hcy-induced neuropathic phenotypes [50].

## Sodium channels

### General overview

Sodium channels are integral membrane proteins that regulate sodium ion levels in cells and the extracellular environment [48]. Sodium is essential for maintaining cellular homeostasis, fluid and electrolyte balance, and blood pressure. Moreover, sodium channels are crucial for the excitability of muscle and nerve cells and for the transport of nutrients and substrates across plasma membranes [118]. In mammals, two major classes of sodium channels exist: voltage-gated sodium channels (VGSCs, Na_v_) and epithelial sodium channels (ENaCs).

VGSCs comprise a family of nine members with relatively uniform fast activation and inactivation kinetics [22, 52]. They generate sodium currents that underlie the initiation and propagation of APs. Different VGSC subtypes are expressed in excitable and non-excitable cells of the central and peripheral nervous system, as well as in skeletal and cardiac muscles. Like other voltage-gated ion channels, including calcium channels, VGSCs possess activation and inactivation gating mechanisms that regulate channel opening and closing in response to changes in membrane potential [61, 97]. Mutations affecting VGSC expression or gating properties can lead to a range of disorders, including myopathies, immune system dysfunction, cardiac arrhythmias, migraines, epilepsy, multiple sclerosis, diabetes, cough, autism, and cancer [34, 61].

ENaCs, in contrast, are primarily expressed in epithelial cells of the kidney, lung, and colon, where they mediate sodium and water transport. As constitutively active channels, ENaCs permit sodium flow from the lumen into epithelial cells across the apical membrane. Their activity is regulated by the renin-angiotensin-aldosterone system and by extracellular factors such as sodium, chloride, protons, shear stress, and proteases [15, 58]. Dysfunctional ENaCs have been implicated in Liddle syndrome, pseudohypoaldosteronism, cystic fibrosis, and may contribute to salt-sensitive hypertension [15]. By mediating electrogenic sodium transport, ENaCs also help maintain transepithelial voltage [1].

### Effects of HHcy on sodium channels

#### Endothelial cells

The influence of HHcy on sodium channels in endothelial cells is largely mediated through ENaCs, as voltage-gated sodium channels are minimally expressed in these cells (Fig. 4; Table 1). Liang and colleagues demonstrated that mouse aortic endothelial cells from animals fed a high-Hcy diet exhibit significant ENaC activation, accompanied by impaired endothelium-dependent relaxation (EDR) [85]. Exogenous application of Hcy to isolated aorta and human umbilical vein endothelial cells produced similar results. Pharmacological blockade of ENaCs with benzamil reversed both ENaC activity and EDR impairment, suggesting a causal relationship between Hcy-induced ENaC activation and vascular dysfunction. These effects were accompanied by elevated ROS levels, linking oxidative stress to Hcy-mediated endothelial dysfunction [53, 85, 124, 130]. Furthermore, cyclooxygenase-2 (COX-2) expression increased in Hcy-treated HUVECs, and the combined presence of ROS and COX-2 is known to enhance vascular smooth muscle hypersensitivity and endothelial dysfunction [109, 122, 140]. Elevated plasma Hcy was also associated with increased thromboxane B2 (TXB2), the stable metabolite of thromboxane A2 (TXA2), which is released via ROS-mediated COX-2 activation [31]. Inhibition of ROS and the COX-2/TXB2 pathway effectively reversed Hcy-induced ENaC activation and EDR impairment, highlighting a potential therapeutic target for HHcy-associated vascular disease [85].

#### Cardiac cells

In cardiac myocytes, HHcy affects both voltage-gated and ENaC-like sodium channels, with pronounced effects on late sodium currents (Fig. 5; Table 1). Cai et al. reported that acute application of pathological concentrations of Hcy to human atrial myocytes significantly increased sodium currents by slowing channel inactivation and promoting recovery, leading to a markedly depolarized resting membrane potential. These effects were reversible upon washout of Hcy [18]. Mouse atrial myocytes from high-Hcy diet animals exhibited enhanced late sodium current (I_NaL_), prolonged AP duration, and increased expression of the Na_v_1.5 channel [56]. The I_NaL_ is a small but persistent current occurring during the plateau phase of the AP. When enhanced, it prolongs AP duration and increases sodium influx, which is accompanied by secondary calcium influx via the Na⁺/Ca²⁺ exchanger. I_NaL_ is implicated in pathophysiological conditions such as heart failure, atrial fibrillation, and myocardial ischemia, and is considered a potential antiarrhythmic target [66, 77]. Cai et al. further demonstrated that Na_v_1.5, together with IP_3_R1, mediates both sodium and calcium influx in response to Hcy, and that knockdown of either protein stabilizes currents and suppresses abnormal electrical activity [54, 56]. While sodium channel dysregulation is clear, some studies still suggest that Hcy does not have a definitive causal association with cardiovascular disease risk [69, 91].

#### Neuronal cells

Voltage-gated sodium channels in neurons are highly sensitive to HHcy (Fig. 6; Table 1). Acutely treated primary cultured caudate nucleus neurons show increased VGSC currents (I_Na_) and a hyperpolarizing shift in the activation-voltage curve in response to Hcy exposure. Upregulation of VGSCs alters intracellular calcium levels and neurotransmitter release, promoting excitotoxicity [41]. Neuroprotective cannabinoids, such as arachidonoylglycerol (2-AG), have been shown to suppress Hcy-induced increases in I_Na_, suggesting potential therapeutic avenues [149]. HHcy affects various neuronal populations, including cortical neurons [84], hippocampal cells [44, 107], caudate nucleus neurons [36], and glial cells [113], contributing to neurological disorders such as epilepsy, Alzheimer’s disease, and dementia [30]. While direct electrophysiological studies of Hcy on neuronal sodium channels are limited, the evidence indicates that sodium channel dysregulation is a major contributor to Hcy-mediated neuronal hyperexcitability.

## Potassium channels

### General overview

Potassium channels represent one of the most diverse and ubiquitously expressed families of ion channels, with over 80 related genes encoding structurally and functionally distinct channels [128]. These channels are typically heterotetrameric complexes, allowing for hundreds of potential configurations. Potassium channels regulate a wide range of physiological processes, including myocardial and neuronal excitability, muscle contraction, neurotransmitter release, and hormone secretion.

Structurally and functionally, potassium channels are categorized into four major types [79]. Inwardly rectifying channels (Kir) consist of two transmembrane segments and seven subfamilies (Kir1-7). Kir channels are constitutively active at resting membrane potential, uniquely allowing inward potassium currents under physiological conditions, and play a critical role in stabilizing negative membrane potentials in excitable and non-excitable cells [71, 105]. Their activity can be modulated by nucleotides, as in Kir6.x channels (ATP/ADP), and by G-proteins or phosphatidylinositol 4,5-bisphosphate (PIP2), as in Kir3.x channels [4, 120, 142]. Kir channels are essential for setting vascular tone in smooth muscle [43], controlling cardiac Aps [74], and generating inhibitory postsynaptic potentials in neurons [88].

Two-pore domain channels (K2P), with two pores and four transmembrane segments, are outwardly rectifying and modulated by various physical and chemical factors, including osmolarity, pH, temperature, and mechanical forces such as stretch or pressure [99]. These non-inactivating channels are active across all membrane potentials and provide background “leak” potassium currents. Fourteen human K2P channels have been identified (e.g., TASK, TREK, TWIK, TRAAK, THIK), contributing to resting membrane potential maintenance and potassium recycling in excitable and non-excitable cells [82].

Voltage-gated potassium channels (VGKCs, K_v_) are modulated by changes in membrane potential and include both inactivating (A-type) and non-inactivating (delayed rectifier) channels [55]. These channels are widely expressed in the CNS, skeletal muscle, and heart, where they shape action potential repolarization and propagation. VGKC channel kinetics determine the duration of AP repolarization, ranging from milliseconds in neurons to hundreds of milliseconds in cardiomyocytes.

Calcium-activated potassium channels (K_Ca_) are outwardly rectifying channels activated by increases in intracellular calcium. They include small-conductance (SK_Ca_), intermediate-conductance (IK_Ca_), and large-conductance (BK_Ca_) channels, which contribute to action potential repolarization and afterhyperpolarization in neurons [127].

### Effects of HHcy on potassium channels

#### Endothelial cells

HHcy alters the function of potassium channels in endothelial cells, contributing to endothelial dysfunction and atherothrombotic disease (Fig. 4; Table 1). Wang et al. reported that intermediate- (IK_Ca_) and small-conductance (SK_Ca_) calcium-activated potassium channels are inhibited by Hcy-induced ER stress in porcine coronary endothelial cells, thereby reducing endothelial function [129]. Large-conductance BK_Ca_ channels, critical for vascular tone regulation, are indirectly disrupted by short-term Hcy exposure in human and rat arterial smooth muscle cells. This inhibition appears to involve NADH/NADPH oxidase-mediated oxidative stress [5, 16, 80], a mechanism consistent with known Hcy-associated ROS generation [130]. In human myometrial cells, Hcy similarly suppresses BK_Ca_ activity by reducing channel expression via a downregulation of the β_1_ regulatory subunit [114]. Sun et al. further demonstrated in porcine coronary endothelial cells that Hcy impairs BK_Ca_ function through downregulation of the β_1_ via ER stress [119]. However, some studies report that long-term Hcy exposure increases oxidized BK_Ca_ channel activity, as observed in rat GH3 cells [49], while short-term application does not alter BK_Ca_ currents, highlighting the complexity of Hcy effects depending on exposure duration.

#### Cardiac cells

In cardiac myocytes, elevated Hcy influences multiple potassium currents, contributing to altered AP dynamics (Fig. 5; Table 1). Cai et al. investigated human atrial myocytes exposed acutely to Hcy and observed inhibition of voltage-gated outward and delayed rectifier potassium currents, possibly through similar mechanisms involving oxidative stress [16, 17]. Interestingly, inwardly rectifying potassium currents were shown to be enhanced upon exposure to Hcy, suggesting a heterogenous regulation of potassium channels by Hcy [16, 17]. Dysregulation of these currents disrupts normal repolarization, contributing to prolonged APs and increased arrhythmogenic risk. Indeed, such changes in potassium channel activity are strongly associated with the pathogenesis of atrial fibrillation, and elevated Hcy levels are recognized as a risk factor for this condition [106, 125]. Through these effects, HHcy-induced potassium channel dysfunction contributes to both electrical instability and increased susceptibility to cardiac arrhythmias.

#### Neuronal cells

Electrophysiological studies on neuronal potassium channels under HHcy exposure are limited (Fig. 6; Table 1). However, evidence suggests that BK channels in neuronal cell lines are sensitive to Hcy. Gaifullina et al., reported that Hcy exposure enhanced BK channel activity in GH3 rat pituitary-derived cells [49]. More importantly, the effect seems to involve a direct redox-sensitive action of Hcy on the channel protein increasing the channel opening probability. Moreover, this effect was observed using inside-out patch-clamp recordings suggesting additional potential intracellular regulatory sites susceptible to interaction with Hcy. Additional insights into neuronal potassium channel effects are inferred from studies in cardiac cells, given the conserved roles of these channels in shaping AP repolarization and regulating excitability. Dysregulation of potassium channel activity in neurons may exacerbate hyperexcitability and contribute to the neuronal pathophysiology observed in HHcy, including seizure susceptibility and altered synaptic signaling.

## Conclusion

Homocysteine has long held a controversial position in biomedical research. During the late twentieth century, the hypothesis that elevated Hcy levels represent a major causal factor in cardiovascular disease was met with substantial skepticism, and proponents of this view were frequently criticized. Over time, however, HHcy has been linked not only to cardiovascular pathology but also to a broad spectrum of neurological disorders, inflammatory conditions, osteoporosis, chronic renal failure, hypothyroidism, insulin-resistant diabetes, polycystic ovarian syndrome, gastrointestinal disorders, and other systemic diseases. In recent years, interest in Hcy biology has re-emerged, and elevated Hcy concentrations are now widely regarded as an independent risk factor for many of these pathologies. Despite these associations, relatively little attention has been devoted to elucidating the molecular mechanisms by which HHcy exerts its detrimental effects on cellular and tissue function.

Ion channels are fundamental membrane proteins that regulate ionic fluxes across cell membranes, thereby governing electrical signaling in the nervous system, excitation-contraction coupling in cardiac and skeletal muscle, and the secretion of insulin and other biologically active molecules. In this review, we synthesize current evidence on the effects of HHcy on ion channel function. Although the available literature provides valuable initial insights, the existing data remain fragmentary and largely descriptive, underscoring the early stage of this field. A comprehensive mechanistic understanding of how HHcy modulates ion channel activity is still lacking, as the mechanisms seem to be rather heterogenous. For instance, Hcy was shown to modulate ion channels through direct redox regulation, but also indirectly by activating receptors and intracellular signaling pathways ultimately altering channel activity and expression, thus contributing to altered ionic homeostasis.

Another important consideration when interpreting the literature is the large variability in homocysteine (Hcy) concentrations used across experimental studies. While physiological plasma Hcy levels typically range between 5 and 15 µM and severe hyperhomocysteinemia rarely exceeds 100 µM, several in vitro studies have used substantially higher concentrations, in some cases reaching the millimolar range. Such supraphysiological levels may produce nonspecific or cytotoxic effects, including oxidative stress or protein modification, which may not fully reflect pathophysiological conditions in vivo. Therefore, results obtained at very high Hcy concentrations should be interpreted with caution when considering their physiological relevance. In addition, different chemical forms of Hcy have been used across studies, including reduced homocysteine, oxidized disulfide forms, and derivatives such as homocysteine thiolactone. These species differ in their chemical reactivity and biological activity, and some derivatives are known to induce specific protein modifications (e.g., N-homocysteinylation). Consequently, the reported effects on ion channel function may depend not only on the concentration of Hcy but also on the specific molecular form used in experimental conditions, which should be considered when comparing studies.

Nonetheless, by integrating the current findings, this review aims to highlight critical knowledge gaps and to encourage further investigations into the ion channel-based mechanisms through which Hcy contributes to disease.

## Data Availability

All data generated or analyzed during this study are included in this published article.

## Abbreviations

AP: Action potential
CBS: Cystathionine beta-synthase
CNS: Central nervous system
COX-2: Cyclooxygenase-2
DRG: Dorsal root ganglia
EA2: Episodic ataxia type 2
EDR: Endothelium-dependent relaxation
ENaC: Epithelial sodium channel
ER: Endoplasmic reticulum
FHM1: Familial hemiplegic migraine-1
Hcy: Homocysteine
HHcy: Hyperhomocysteinemia
HVA: High-voltage-activated
IP3: Inositol 1,4,5-trisphosphate
IP3R1: Inositol 1,4,5-trisphosphate receptor type 1
K2P: Two-pore domain potassium channel
KCa: Calcium-activated potassium channel
Kir: Inwardly rectifying potassium channel
LGCC: Ligand-gated calcium channel
LVA: Low-voltage-activated
MTHFR: Methylenetetrahydrofolate reductase
NMDA: N-methyl-D-aspartate
NO: Nitric oxide
PIP2: Phosphatidylinositol 4,5-bisphosphate
ROS: Reactive oxygen species
SCA6: Spinocerebellar ataxia type 6
TXA2: Thromboxane A2
TXB2: Thromboxane B2
VGCC: Voltage-gated calcium channel
VGKC: Voltage-gated potassium channel
VGSC: Voltage-gated sodium channel
VSMC: Vascular smooth muscle cell
